# Engaging patients and the public in Health Research: experiences, perceptions and training needs among Manitoba health researchers

**DOI:** 10.1186/s40900-019-0162-2

**Published:** 2019-10-08

**Authors:** Leah K. Crockett, Carolyn Shimmin, Kristy D. M. Wittmeier, Kathryn M. Sibley

**Affiliations:** 1 Department of Community Health Sciences, Rady Faculty of Health Sciences, University of Manitoba, 374(1) – 753 McDermot Avenue, Winnipeg, MB R3E 0T6 Canada; 2 George and Fay Yee Centre for Healthcare Innovation, 379 – 753 McDermot Avenue, Winnipeg, MB R3E 0T6 Canada; 3 Department of Physiotherapy, Winnipeg Health Sciences Centre, RR132 - 820 Sherbrook St, Winnipeg, MB R3A 1R9 Canada; 4 Department of Pediatrics and Child Health, Rady Faculty of Health Sciences, University of Manitoba, 715 McDermot Avenue, Winnipeg, MB R3E 3P4 Canada

**Keywords:** Patient and public engagement, Cross-sectional survey, Health research

## Abstract

**Background:**

The significance of patient and public engagement is increasingly recognized in health research, demonstrated by explicit requirements for patient and public engagement by funding agencies and journals. Such requirements have charged health researchers with leading patient and public engagement efforts, but evidence suggests that this practice is still evolving. Little research has explored the experiences and training needs of health researchers. This study aimed to establish a baseline understanding of the experiences, perceptions and training needs of health researchers in engaging patients and the public in health research in the context of Manitoba.

**Methods:**

A cross-sectional 50-item questionnaire was distributed using a multi-phase purposive sampling strategy targeting health researchers in Manitoba, Canada. Data was summarized using frequencies, percentages and analyzed using chi-square testing. A local patient engagement advisory group was consulted at the interpretation stage of the study to obtain feedback and input on the findings and their implications.

**Results:**

Responses from 53 health researchers were included. Most participants had engaged patients and the public in their own research (*n* = 43, 81.1%). Those who had engaged reported having some (*n* = 19, 44.2%), extensive (*n* = 14, 32.6%) or a little (*n* = 10, 23.3%) experience with this process. Most engaged at the levels of inform, consult or involve (81.3, 64.6 and 54.2% respectively), while fewer engaged at the collaborate (37.5%) or patient-directed levels (12.5%). Recruitment occurred using a number of approaches and engagement occurred at various phases of the research process, while main groups engaged were patients (*n* = 38, 82.6%) and families/caregivers (*n* = 25, 54.4%). Barriers to engaging patients and the public in health research included funding, time, compensation, logistics, recruitment, motivation at both the patient and researcher level, and skills of researchers to engage. Researchers reported an overwhelming need and interest for supports, funding and training to effectively engage patients and the public in health research. Consultation with the patient advisory group provided further insight on study findings and areas for future research.

**Conclusions:**

Participating Manitoba health researchers engaged patients and the public in health research at multiple, but typically lower levels of involvement. Findings highlight the barriers to effective, authentic and meaningful patient and public engagement and support the need for targeted training, supports, funding and time for health researchers.

## Plain language summary

Engaging patients and the public in conducting research is recommended and becoming more common in health research. However, engaging patients and the public in research is a new skill for many researchers. Understanding the experiences, views and education needs of health researchers can help inform the development of training strategies and resources. In this study, we surveyed researchers from Manitoba (Canada). Most researchers who answered our survey had engaged patients and the public within the research process at some point in their career. Overall, researchers recognized the important role of patients and the public within the research process and engaged them in many ways. Despite this, we found overall low levels of engagement with the public (for example: only “telling” them about their research or asking for their opinion, rather than having them as shared partners). This study found that researchers face challenges in both engaging patients and the public in health research and also in undergoing training. These challenges included; time, university responsibilities, funding, motivation and skills. Researchers expressed an overwhelming desire for further training. We also heard from a local patient research advisory group about their perspectives of the findings. Their comments gave us more insight into the study findings and suggested topics for future research. This study highlights challenges that researchers face when involving patients and the public in their work and potential training and resources needed to fill these gaps.

## Background

The Canadian Institutes of Health Research (CIHR) defines patient engagement in health research as the meaningful and active involvement of patients in the governance; priority setting; conduct; and translation of research [1]. While various contexts use different terminology to describe the engagement process, such as patient and public involvement (PPI) in the UK, the term patient and public engagement has largely predominated in North America and in the context of this study, the province of Manitoba, Canada. Despite varying terminology, the concept of patient and public engagement has received growing national and international attention and both the benefits and challenges of stakeholder engagement in the research process are increasingly recognized. Similarly, there are increasing demands on researchers to demonstrate patient and public engagement within the research process to funding bodies and journals. However, there is ongoing concern that current efforts are often tokenistic [2] and aimed at fulfilling requirements for funding applications and journals, rather than placing a true value on the engagement of patients and the public in the research process.

There are a number of research engagement roles for patients and the public. The International Association of Public Participation (IAP2) spectrum is used actively across Canada, Australia, New Zealand, Indonesia, Italy, Southern Africa and the USA to outline levels of engagement and promote best practices in patient and public engagement. The IAP2 spectrum ranges from the lowest level of engagement known as “inform”, which involves letting patients and the public know about research findings, to “consult”, where the goal is to obtain feedback from patients and the public, to “involve”, where researchers work directly with patients and the public and share the decision-making power, and finally to the highest level of engagement known as “empower”, where all decision-making is in made by patients or the public, who actively control, direct and manage the entire research process [3]. Engaging patients and the public at any level of the spectrum requires distinct knowledge, attitudes and skills, and may represent a new paradigm for some health researchers. However, there is evidence suggesting that this practice is still evolving [4–6]. For example, a modified Delphi study conducted in the UK in 2014 examined the opinions of members of the public, researchers and research managers, regarding areas of consensus and conflict, barriers, drivers, and perceived impacts of patient and public engagement in health research [7]. Lack of training, funding and time, along with attitudes and perceptions of researchers were among the identified barriers for effective engagement [7]. Key recommendations from this work supported the need for clearer guidance on the purpose of and measurable standards of patient and public engagement, more financial and educational support from institutions and funders for both researchers and patients and the public, and the need to redress power imbalances between researchers and patients and the public.

Yet, while the emphasis on patient and public engagement in research is expanding, training in patient and public engagement has received little research attention and it is believed that funding and publication requirements have outpaced evidence about the practical aspects of patient and public engagement [5, 8, 9]. A 2015 qualitative study examining the views of both researchers and patients and the public found mixed views on researchers needs for training in the field [5]. Though most researchers had either received training or indicated that they would find it helpful, others had practical experience and felt that there was insufficient evidence to inform training [5]. Their findings suggested that in order to provide training and improve uptake, there is a need to better understand and articulate what both researchers and contributors can expect to gain from training in patient and public engagement [5]. Furthermore, findings indicated a further need to consider how training is conceptualized, designed, promoted and delivered in order to enhance its uptake and relevance [5].

The current study aimed to address the above noted gaps relating to experiences, perceptions, and training needs in the context of the Canadian province of Manitoba. Understanding local context is a recommended component of implementation process frameworks [10] and can provide important insights that can inform the development of interventions to facilitate the practice of engaging patients and the public in health research, and may be applied in similar settings. Manitoba has a population of approximately 1.2 million, with four public universities conducting health research. The goals of this study were: (1) to establish a baseline understanding of Manitoba researchers’ current experience, knowledge and perceptions relating to engaging patients and the public in health research, and (2) to identify needs and training for supporting researchers in engaging patients and the public in health research.

## Methods

### Study design

An online cross-sectional survey was developed and distributed to health researchers in Manitoba, Canada using a multi-phase purposive sampling strategy. The study investigators are affiliated with Canada’s Strategy for Patient Oriented Researcher (SPOR), a national initiative between patients, researchers, healthcare providers and decision-makers to promote patient-oriented research, a better healthcare system, and better health outcomes. SPOR Support units exist across all provinces and territories. The study team, including Manitoba SPOR unit patient engagement and knowledge translation leads, and a clinican scientist, were involved in the conceptualization, design, analysis and interpretation phases of the study. Furthermore, a local patient and public engagement group was launched 2 years later, and therefore, was engaged in the data interpretation phase.

### Questionnaire instrument

A custom questionnaire was developed for the study. The questionnaire contained 50 items designed to explore the above noted objectives. Questions relating to *experience* included questions relating to knowledge (3 items), overall experience (9 items) and barriers and facilitators (1 open-ended question), while questions relating to *perceptions* included questions relating to self-efficacy (9 items) and attitutes). Finally, the remaining questionnaire items explored the *training* needs (6 items) and demographic information (5 items) of respondents (see Additional file 1). Survey development was informed by adapting aspects of both the Theoretical Domains Framework (10) and the Determinants of Implementation Behavior Questionnaire (11). The survey format included check boxes, ranking and open-ended questions and participants were diverted to various sections of the survey based on previous engagement or not. The survey was piloted by a convenience sample of 3 health researchers across domains of health research to evaluate clarity, ease of administration, time to completion and potential missing information. Pilot data were not included in the final analysis.

### Participants and recruitment

Health researchers in the province of Manitoba, Canada were eligible to participate. A health researcher was defined as someone who spends at least 10% of their working time conducting independent health research [11] and who held a faculty appointment at a Manitoba University or was eligible to apply for competitive health research funding as primary applicant. A multi-phase, purposive sampling strategy was used, targeting health researchers from four provincial academic institutions and those who identified as being eligible as per the criteria questions. In phase I, participants were invited to participate via email advertisement circulated through institutional distribution lists over three consecutive weeks. The distribution lists reach over 400 individuals; however, these are not specific to independent researchers so it is not possible to know how many eligible individuals were invited. Based on results from the initial recruitment, we conducted a second phase of recruitment using a targeted recruitment strategy and updated messaging to gain broader representation from those who had little to no experience engaging patients and the public in health research. Our second targeted strategy included directly contacting key communication specialists and use of social media platforms to enhance recruitment. Conservatively estimating that 20% of respondents would report needing support to more effectively engage patients and the public in their research, a sample size of 61 was deemed necessary to achieve adequate power at a 95% confidence level with 80% precision [12].

### Data collection and storage

Survey responses were collected and stored through FluidSurveys™ in phase I and SurveyMonkey in phase II. Due to a cease in operation of FluidSurveys™, data from phase I were extracted, placed in a password protected file, and merged into the new survey platform, SurveyMonkey. No changes were made to survey format or questions between phases, with the exception of an additional demographic question exploring respondents’ primary research “pillar”, a categorization system developed by CIHR that includes biomedical, clinical, population health, and health services research .

### Data analysis

Raw and summary data were exported into Excel. Respondents with less than 80% completion were removed from the analysis. Data with categorical responses were summarized using frequencies and percentages. Where applicable, responses were stratified by those who reported previously having some or extensive experience engaging patients and the public in health research (*n* = 33, 62.3%) and those who had none or little experience (*n* = 20, 37.7%) and responses were compared using chi-square testing, with significance level set to *p* < .05. Due to small cell counts, the 5-point Likert scale responses of “strongly agree” and “agree”, and response options of “strongly disagree” and “disagree” were collapsed to form “agree” and “disagree”. Similarly, 5-point Likert scale responses of “some” and “a little” and “not at all” and “don’t know” were collapsed to “somewhat” and “not at all”. Measures of self-efficacy were ranked using a Likert scale from 1 (strongly disagree) to 10 (strongly agree). Codes and themes were developed for open-ended responses.

### Patient and public engagement in data interpretation

To facilitate data interpretation and outline future directions for researcher support and training, we engaged at the *consult* level with the George & Fay Yee Centre for Healthcare Innovation’s (CHI’s) Patient and Public Engagement Collaborative Partnership (“the Partnership”) during the interpretation stage of this study. The Partnership includes a diverse group of individuals who work with CHI and health researchers to co-create and advise on authentic engagement strategies, health research policies, resources, tools, services and programs, and who engage with various researchers throughout many stages of the research process. Planning for the engagement process within this study was guided by an appreciative inquiry approach [13]. A user-friendly summary outlining the project purpose and results was distributed to committee members 3 weeks in advance of a regular quarterly meeting, while the questionnaire and manuscript tables were provided 1 week prior to the meeting for further detail. This was followed by an in-person presentation by one member of the research team (LKC) at the meeting, providing further opportunity for interpretation, feedback and discussion. Committee members were provided 3 weeks to provide any additional insight following the quarterly meeting. General themes were summarized by the study team. Finally, the study team followed-up with the committee by distributing a summary outlining how their feedback was incorporated into the manuscript, and by circulating the final manuscript. The Partnership was only engaged in the interpretation stage of the study.

## Results

### Participants

Seventy five individuals responded, including 35 in phase I and 40 in phase II. Fourteen declared that they were not eligible, leaving 61 respondents eligible to participate. Of these 8 completed less than 80% of questions and were removed from the analysis. The final analysis includes data from 53 health researchers. Table 1 presents participant demographics for the entire sample. Overall, early-career researchers (*n* = 21, 39.6%) and mid-career researchers (*n* = 19, 35.9%) responded more than established researchers (*n* = 13, 24.5%). Most participants conducted research in the university setting (*n* = 33, 62.3%) and used quantitative (*n* = 23, 43.4%) or mixed methods (*n* = 22, 41.5%) as their primary research methodology. Included as a question part way through phase I and all of phase II, respondents were more likely to identify the CIHR Health Systems and Services (*n* = 15, 39.5%) and Population and Public Health (*n* = 13, 34.2%) health research pillars. Overall, most participants had engaged patients and the public in their own research (*n* = 43, 81.1%) and these respondents reported having some (*n* = 19, 44.2%), extensive (*n* = 14, 32.6%) or a little (*n* = 10, 23.3%) experience with this process. Other participants reported no experience engaging patients and the public within the research process (n = 10, 18.9%).

**Table 1:** Characteristics of Respondents

Characteristic	Entire Sample (*n* = 53) n (%)
Career stage	
Early-career researcher (≤ 5 years)	21 (39.6%)
Mid-career researcher (16–15 years)	19 (35.9%)
Established researcher (16+ years)	13 (24.5%)
Primary research setting	
University	33 (62.3%)
Research Institute	5 (9.4%)
Hospital	9 (17.0%)
Other	6 (11.3%)
Primary research methodology	
Quantitative	23 (43.4%)
Qualitative	8 (15.1%)
Mixed-methods	22 (41.5%)
Health research pillar	
Health Systems and Services	15 (28.3%)
Population and Public Health	13 (24.5%)
Biomedical	5 (9.4%)
Clinical	5 (9.4%)
*Missing*	15 (28.3%)
Previous engagement experience	
Little or no experience	20 (37.7%)
Some or extensive experience	33 (62.3%)

### Subgroup analysis

We conducted a subgroup analysis which stratified participants by those with some or extensive engagement experience (*n* = 33, 62.3%) and those with little or no engagement experience (*n* = 20, 37.7%). We found no significant differences between the two groups by demographic characteristics, as outlined in Table 1.

### Experience

Among those who reported ***any*** prior engagement experience (little, some or extensive), the levels of engagement varied. Based on the established IAP2 levels, and allowing respondents to select all that apply, most engaged at the levels of inform (*n* = 36, 83.7%), consult (*n* = 28, 65.1%) or involve (*n* = 22, 51.2%), while fewer engaged at the levels of collaborate (*n* = 16, 37.2%) or patient-directed (*n* = 5, 11.6%). Notably, this and subsequent questions within this section were only asked of those who had ***any*** engagement experience (*n* = 43), excluding those with no prior experience (*n* = 10).

Researchers who had engaged were most likely to engage with patients (*n* = 34, 79.1%) and families/caregivers (*n* = 23, 53.5%), followed by community organizations (*n* = 20, 46.5%) or specific patient or health issue organizations (*n* = 19, 44.2%), the general public (*n* = 15, 34.9%), geographic community (n = 10, 23.3%), and others (n = 3, 7%) in a check all that applies question. Patients and the public were recruited using a number of approaches, and were engaged all throughout the research process – from the earliest stages of priority-setting right through to dissemination – but there was no phase of engagement that all respondents had a unified level of experience with. The least commonly involved phases were grant proposal writing and data analysis.

#### Health researchers with no experience

Among those who had not engaged, the primary reasons for not doing so included resources (*n* = 4, 40%), inexperience (n = 4, 40%), lack of training and organizational directive for their inclusion (n = 4, 40%), uncertainty as to whether or not patients should be involved in their research (n = 4, 40%), and time (*n* = 3, 30%). The majority of these participants felt that they did not have a good understanding of the field of patient and public engagement (*n* = 9, 90%).

### Perceptions

This section reports on all participants (*n* = 53) as a whole. Overall, participants felt moderately equipped with the knowledge (average: 6.0/10) and skills (average: 6.4/10), associated expectations (average: 6.0/10), and relative appropriateness (average: 6.6/10) for engaging patients and the public in their research. Those who reported some and extensive engagement of patients and the public in their research were more likely to express confidence in their ability to engage patients and the public in the research process (53.1% vs 20.0% agreement, X^2^ = 5.61, *p* = 0.02) and were more likely to feel comfortable exploring sensitive topic areas (54.8% vs. 20.0%, X^2^ = 6.09, *p* = 0.01) compared to those who had little or no engagement experience.

Participants’ attitudes and beliefs on the value, process and outcomes of engaging patients are reported in Table 2. With regards to general statements about the value of engagement, participants overwhelmingly agreed with the importance of engagement (90.4%), the value of its contribution to research (80.8%) and the healthcare system (86.5%), the right of patients and the public to be engaged (82.7%), and its role as an integral part of patient-oriented research (88.5%).

**Table 2:** Attitudes and beliefs with regards to engaging patients and the public in health research

Question	Level of agreement (*n* = 52) n (%)			
	Disagree	Neutral	Agree	*Missing*
General				
Engaging patients and the public in health research is important	2 (3.8%)	3 (5.8%)	47 (90.4%)	*-*
Engaging patients and the public in health research can improve the value of research	4 (7.7%)	4 (7.7%)	42 (80.8%)	*2 (3.8%)*
Engaging patients and the public in health research can improve the value of the healthcare system	1 (1.9%)	4 (7.7%)	45 (86.5%)	*2 (3.8%)*
Patients and the public have a right to be engaged in health research	4 (7.7%)	3 (5.8%)	43 (82.7%)	*2 (3.8%)*
Engaging patients and the public in health research is an integral part of patient-oriented research	1 (1.9%)	3 (5.8%)	46 (88.5%)	*2 (3.8%)*
Engaging patients and the public in my own research is useful	2 (3.8%)	3 (5.8%)	45 (86.5%)	*2 (3.8%)*
Personal				
Patient and public engagement is compatible with my program of research	3 (5.8%)	9 (17.3%)	38 (73.1%)	*2 (3.8%)*
Engaging patients and the public in my research will interject bias in my research	32 (61.5%)	8 (15.4%)	11 (21.2%)	*1 (1.9%)*
I feel pressured to engage patients and the public in my research	26 (50.0%)	10 (19.2%)	13 (25.0%)	*3 (5.8%)*
I feel confident in my ability to engage patients and the public in my research	14 (26.9%)	17 (32.7%)	21 (40.4%)	*-*
I feel comfortable to explore sensitive topics with patients and the public	15 (28.8%)	16 (30.8%)	21 (40.4%)	*-*
Current state of patient and public engagement in health research				
Patients and the public are actively and meaningfully engaged in health research	16 (30.8%)	15 (28.9%)	19 (36.5%)	*2 (3.8%)*
Funding agencies provide sufficient financial reimbursement to researchers to engage patients and the public in health research	26 (50.0%)	17 (32.7%)	8 (15.4%)	*1 (1.9%)*
My institution values engaging patients and the public in health research	8 (15.4%)	15 (28.9%)	27 (51.9%)	*2 (3.8%)*

With regards to engagement within respondents’ specific research programs, most agreed that patient and public engagement was compatible with their research program (73.1%). They also disagreed with statements about feeling pressured to engage (50.1%) or about concerns for its subsequent contribution to bias within their research (61.5%). When considerindering the current state of meaningful engagement overall in health research, responses were more varied. Fewer than half of respondents agreed that patients and the public are being meaningfully engaged in research (36.5%), while only 15.4% of respondents felt that financial reimbursement to researchers by funding agencies was sufficient. Furthermore, just over half of respondents felt their institutions valued engagement in research (51.9%).

Most respondents disagreed when asked if there were areas where it might not be appropriate to engage patients and the public in health research (*n* = 38, 71.7%). Among those who did indicate areas where it might not be appropriate in an open ended question (*n* = 15, 28.3%), these related mainly to basic and biomedical research and in situations where the risks to patients might outweigh the benefits. Participants also stressed the need for careful consideration when engaging with vulnerable populations. Others focused on the appropriateness of patient and public engagement based on level of involvement, stages of involvement in the research process, and based on logistical factors such as time, practicality and geographical limitations (Table 3).

**Table 3:** Areas where inappropriate to engage

Themes	Representative quote
Basic and biomedical research	“I’m not sure how basic/bench sciences are incorporating these concepts, but I think there is merit to engage patients in those areas also, as it will help with the overall populations education and knowledge about health treatments (especially for example in immunization knowledge)” “Some basic science questions they are not equipped to provide guidance on” “Basic sciences where extensive training and knowledge is required”
Vulnerable populations	“If appropriately developed, there is no area that could not be appropriately considered as a research focus. Children and the elderly comprise a considerable impediment due to informed consent issues.” “Extra caution must be taken with vulnerable populations”
Harm vs. benefit	“Research that involves potential risk for biased input in developing and disseminating the research and outputs e.g., due to fear, popular opinion, etc.” “… where there will be more harm than benefit – case by case, not necessarily any particular area of research”
Level of involvement	“I’m sure there are, but this will depend on the level of engagement” “I’m not sure that it is inappropriate ever to engage patients if there is sufficient time taken for adequate education about the research methods and process - the expert role is to know and outline the pros and cons of research decisions throughout the process so the engaged team can decide; knowledge is power and will help our society as a whole.”
Research phase	“I don’t think having patients set the agenda is always appropriate. The skills needed for good research is with researchers, and spending money on whimsical subjects with no clear outcome is wasteful.” “I’m not sure it’s always appropriate to include patients in research design and analysis phases” “… should occur at arms’ length from the research. They are not formally trained in research methodology. While their insight may be valid at onset (design) or interpretation of findings, I would hesitate to involve them beyond that.”

### Health researcher needs for supports and training in patient and public engagement

Most participants reported needing more supports to effectively engage patients and the public in their research (*n* = 43, 81.1%); 95.0% of those who had little or no engagement experience, and 74.2% among those who had some or extensive experience. Health researchers reported needing advice or guidance to plan the process (*n* = 18, 41.9%), support to facilitate the engagement process over time (*n* = 15, 34.9%), and access to a resource such as a website (n = 15, 34.9%). Others highlighted the need for funding to develop community partnerships and more generous financial support and timelines given the intensive and sometimes lengthy process of engagement.

The majority of participants indicated that they would participate in patient and public engagement training if it were available (*n* = 40, 75.5%). Reasons for lack of interest in training included lack of time (*n* = 23, 62.2%), the unavailability of training opportunities (*n* = 17, 46.0%), and being unsure whether it applied to their research (*n* = 10, 27.0%). Other open-ended responses included competing priorities such as heavy teaching loads and training that was too general in nature to support broad multiple stakeholder engagement or to be inherently appealing to health researchers. The most frequently-identified topics of interest for training included approaches to engagement (*n* = 33, 82.5%), planning for patient and public engagement (*n* = 28, 70.0%), recruiting for engagement (*n* = 25, 62.5%), supporting on-going engagement and addressing challenges to engagement in research (*n* = 24, 60.0%), and engaging and including specific populations, such as youth, diverse communities, and hard to reach populations (n = 24, 60.0%). Open-ended responses included developing research questions and proposals with patients and the public, the role of pharmaceutical companies in clinical research, the ethics of industry-sponsored research, and engaging children in research.

### Barriers to engaging patients and the public in health research

Overall, participants identified a range of perceived barriers to engaging patients and the public in health research, including funding, time, compensation and logistics, recruitment, motivation at both the patient and researcher level, and skills of researchers to engage (Table 4).

**Table 4:** Barriers to engaging patients and the public in health research

Barrier	Participant example
Funding, time and compensation	- “Funding to have adequate support” - “Funding for meetings with them” - “Funding sufficient to complete a project with sufficient power to complete the research problem” - “Their time. When we ask patients/public to engage, it is usually as a volunteer” - “Parking access, time required for participation, unable to offer much incentive” - “Lack of adequate compensation for time and training” - “The expensive and time consuming nature of engaging patients and the public in health research” - “Mismatch between funding timelines and fieldwork time needed to engage marginalized and vulnerable populations” - “Graduate studies timelines required to do true community based research”
Skills and motivation	Patient - “Interest by the patients/community in my research/area of research” - “Increasing motivation and excitement from patients/public for the project” - “Long term relationships are difficult as people move from small grassroots organizations readily. My advisory group is an ever-changing, ever-growing group. A few people clearly provide ongoing, regular constant involvement and they have become part of a smaller steering committee.” Researcher - “Community organizations want researchers to support their programs/activities, even when research findings suggest a more complex position on a topic. Difficulty finding participants who are interested in more than brief interactions.” - “Wondering how patients would understand the value of my research” - “My own lack of training/steep learning curve. I kind of muddle through”
Recruitment	- “Access to suitable patients from clinics” - “recruitment (costs, success in reaching appropriate numbers) is often a problem - time-consuming making phone calls - sometimes difficult for older adults to access University campus” - “Finding patient participants with direct experience with treatments that are not yet available in Canada.”
Logistics	- “Engaging patients and public can be expensive and time consuming; often timelines for using funding and providing reports seems too short for the fieldwork needed to engage patients/public, especially with marginalized and vulnerable populations. Graduates studies timelines also can make it impractical to do true community based participatory research. I think funding is slowly getting easier to provide support for time needed to do engagement research, but it is a process.” - “Time, practical factors such as geographical limitations” - “Time, space, clear goals, good representation, continuing involvement, good merit” - “Timing and location” - “Privacy legislation”

### Funding, time and compensation

The most frequently reported systemic barrier related to insufficient funds and time allocation within grants to appropriately and meaningfully engage with patients and the public. One respondent noted the expensive and time-consuming nature of engaging patients and the public in health research, and the mismatch between funding timelines and fieldwork time needed to engage marginalized and vulnerable populations. Another respondent noted that graduate studies timelines for their trainees can also make it impractical to do true community based participatory research. Funding to have adequate support to hold meetings, to compensate patients for their time and expenses such as parking, and lack of adequate compensation for time and training were also noted. Respondents noted that when asking patients and the public to engage, it was usually as a volunteer with the inability to offer much incentive for their time.

### Skills and motivation

Individual barriers were related to motivation by both the researcher and patients and the public, and skills of the researcher. Researchers noted a need to enhance interest, motivation and excitement from the patients and communities in their own research. Another researcher spoke of the difficulty in building and maintaining long-term relationships given ongoing turnover in small grassroots organizations and advisory groups, and the difficulty for patients and the public to provide ongoing, regular and constant involvement.. From the researcher point of view, one researcher wondered how patients would understand the value of their research while others noted that programs and activities offered by community organizations are often contrary to research findings. Finally, one respondent spoke of their own lack of training in the area and the steep learning curve required to engage patients and the public.

### Logistics

Logistical barriers included practical barriers such as geographical limitations, ways to determine appropriate timing and location for engagement, and issues with recruitment. Finally, the need for clear goals, adequate representation, continuing involvement and good intentions were noted.

### Patient and public engagement collaborative partnership input

The above noted results were of interest to the committee members of “the Partnership” (*n* = 5), who initiated discussion and provided feedback about study design, academic processes and results while interpreting study findings.

First, the committee highlighted our definition of a “health researcher”, which potentially excludes specific voices. Notably, they expressed that academic health sciences researchers are often those who struggle most with engaging throughout the research process compared to those who conduct practice-based, and often unpublished research “on the ground” in settings such as northern communities and community based organizations. Given the narrow focus of our definition of a health researcher, the committee suggested that follow-up surveys target a broader range of participants. They also suggested re-distributing the survey to the same audience given advances in the field of patient and public engagement since this survey was first distributed to examine whether perspectives have shifted. The Partnership also discussed issues with systemic processes and funding criteria within academia, particularly tri-council funding, and the importance of developing non-traditional research processes (ie. an incorporation to hold funds) to facilitate the conduct of research with or among non-academics, such as community researchers, and patients and the public.

Based on their own experiences being engaged within the research process, the Partnership noted the importance of following up with patients and the public post-engagement to provide information on how their views were incorporated into the final research product. Therefore, a suggested addition to the survey were questions regarding whether or not researchers had followed up with patients or communities following engagement, and their process for doing so. Given low levels of engagement during the grant proposal and data analysis phase, committee members discussed challenges with their own experiences being engaged at these phases. This included providing insufficient information to equip patient partners to provide effective feedback, lack of context, and pre-conceived notions that patient partners might not have the technical experience or interest to view entire applications. Finally, they felt that some findings within this study seem to indicate that researchers are not always seeing the value of patient and public partner perspectives in research, despite successful engagement experiences from the experiences of this committee in the past. However, they acknowledged that these perspectives may have shifted over time.

While some members of the committee expressed the need for researchers to relearn how to involve and speak the same language as patients and the public, others disagreed and expressed the need for both sides to train together and meet in the middle to allow for meaningful engagement. Furthermore, the Partnership encouraged the promotion of patient and public engagement early within graduate program curriculums to encourage thoughtfulness around patient and public engagement from the earliest stages of research design, particularly given the finding that 90.0% of those who had never engaged did not have a good understanding of the field of patient and public engagement. Finally, the Partnership felt that the findings presented by the research team in this study could not only inform future research, but also help determine future directions and priorities for the committee.

## Discussion

Despite the growing practice of engaging patients and the public in health research, few studies have examined the experiences, perceptions and training needs of health researchers. This study presents several key findings,information to guide the development of appropriate support for engagement, and future research directions that will be required to advance patient and public engagement within the local context.

This study supports an overwhelming need for more resources and supports to adequately equip health researchers to engage patients and the public in research.. This is consistent with the emerging literature in this area which suggests a need for frameworks, training and supports for researchers conducting patient and public engagement within their programs of research, not just the patriarchal view of educating patients and the public who wish to engage [5, 8, 14–17]. Similarly, past research has acknowledged the competencies required to meaningfully engage patients and the public in health research, but the lack of associated training available for researchers (19). Most participants expressed interest across a range of topics, indicating a broad need for support. However, several barriers, such as other academic responsibilities and expectations, time, and preferences for training content, could limit health researchers from accessing training were it available. Previous studies have noted similar barriers to engaging patients and the public within the research process generally (7, 19). Results from this study also provide a better understanding of the landscape of patient and public engagement among health researchers across the province. We found most researchers engaging at the lower levels of inform, consult or involve, which increasingly do not fulfill requirements of funding agencies and journals, and fewer engaging at the levels of collaborate or empowerment. Similarly, the self-efficacy and confidence of health researchers to engage patients and the public increased with experience. For example, researchers who had more experience engaging were more likely to know when it was appropriate to do so, and were less likely to report feeling discomfort exploring sensitive topic areas. This suggests a need to consider the career stage and patient and public engagement experience of researchers when developing training and resources, as there is not likely to be a “one-size fits all” solution. Mobilizing the expertise of more experienced researchers in training and resource development is one potential strategy that could be explored through peer-to-peer mentorship or other approaches.

There was high agreement among researchers about the importance, value and usefulness of patient and public engagement in health research. Yet, despite these positive views, many felt that patients and the public were not being meaningfully engaged, nor that there was sufficient funding or institutional value to allow researchers to actively and authentically engage. This is reflected further by the systemic barriers reported within the study, which include a lack of adequate funds and time allocation within grants to conduct appropriate engagement. These reported barriers move beyond training to further advocacy at the institutional and funding levels.

Individual doubts and barriers noted within the study, including difficulties maintaining relationships, aligning values, the ongoing mismatch between research and practice, and researchers own skills in engaging patients and the public in health research provide insight into areas which could be address through training. Notably, several of these barriers may not be unique to patient and public engagement, allowing us to potentially draw from other fields. For example, relationship maintenance is crucial to the success of any collaborative research (20). Nonetheless, it can be argued that engaging with patients and the public presents a distinct and unique cultural shift from traditional forms of *most* academic research and requires more time, resources and additional skills (5). Additionally, approximately one-third of respondents indicated areas where it may not be appropriate to engage patients and the public within research, which varied based on level of engagement, stage of the research process, type of research, and other factors such as time, practicality and geographical limitations. Based on these findings, specific training and resources around engagement in biomedical research, the various levels of engagement and ways to engage, and the use of trauma-informed approaches (21) appear to be important avenues for further focus and training.

Although it was difficult to recruit those who had never engaged, the emerging findings provide insight into the characteristics, perceptions and barriers to patient and public engagement within this subset of health researchers. General research findings demonstrate that majority of those who had never engaged were early career researchers conducting quantitative research, although these differences were not statistically significant. This observed pattern may be reflective of the nature of the early stages of an academic career, where publication, productivity and tenure are high priority and often in mismatch with the time needed to conduct meaningful stakeholder engagement (22). Again, the findings suggest that the majority of those who had engaged were mid-career researchers conducting mixed-methods research, a trend which has been reported previously (23) and is not surprising given the emphasis on both the *what* and *how/why* among mixed-methods researchers. This may reflect the emergent timing of the growing international movement towards patient and public engagement in accordance with the timing of their training, and also a greater level of comfort in their academic security post-tenureship. Notably, the majority of those who had little or no engagement experience felt they did not have a good understanding of the field of patient and public engagement, confirming the need for additional training and resources.

Finally, we consulted patients and the public within the interpretation stage of this project. Overall, patients and members of the public were receptive and resonated with the results of this study. However, several study design and researcher beliefs were pinpointed by the Partnership. For example, the definition of a health researcher and subsequent inclusion criteria – an individual who spends at least 10% of their time conducting independent health research or who is eligible to apply for competitive funding as a primary applicant – was troublesome for the committee, who viewed community members and people with lived experience as knowledgeable entities within the research process who should have been included in this study. This is a direction for future research to ensure inclusion of a more encompassing group of researchers, given appreciation of alternate ways of knowing and the emergence of integrated knowledge translation in health research (24, 25). Furthermore, the Partnership discussed the concept of meaningful engagement and the need to incorporate additional methods, such as interviews, in the future to truly capture whether patients and the public are being meaningfully engaged within the research process.

## Limitations

The responses received in this survey reflect the experiences of a subset of health researchers within Manitoba, Canada, which may differ from other provinces within Canada and influence the interpretation of the findings. However, given knowledge translation theory [10] and the purpose of the current study, gaining a more in-depth understanding of local context was an important component of developing health researcher training. Furthermore, the survey relied on self-report measurements, which may overestimate perceived performance of a behaviour. As noted previously, despite our attempts to gain a greater response rate from those who had not previously engaged patients and the public in health research, we received only 10 responses from these researchers. To better balance our sample size, we combined those with no or little experience to balance the sample size between the two groups. However, our results still favor the views and experiences of researchers with more experience engaging patients and the public in health research. Furthermore, despite our attempts to reach an adequate sample size, we fell short of our original target. Although our questionnaire development was *informed* by validated instruments – the e Theoretical Domains Framework [18] and the Determinants of Implementation Behavior Questionnaire [19] – it is important to note that we did not use a validated instrument to conduct this survey. Finally, although involvement of patients and the public within the entire research project would have been ideal, we were only able to engage at the consult level at the interpretation stage of our analysis, as this committee was not established during the early study phases. Nonetheless, this exercise provided valuable additional input from the patient perspective and future directions for research, practice, and training.

## Implications

The results of this study confirm the need to develop and provide training for health researchers in the area of patient and public engagement. Additional input from the Patient and Public Engagement Collaborative Partnership allowed us to incorporate feedback and identify potential study limitations, and future research directions. With increasing funding and publication requirements for patient and public engagement and the associated implications for health research, paying greater attention to the needs of researchers to authentically engage within the research process will benefit the conduct and translation of meaningful patient-informed research into practice.

## Conclusions

This study is among the first to examine the experiences, perceptions and training needs of health researchers to engage patients and the public in health research. The majority of our Manitoba sample reported engaging patients and the public in their research programs and expressed positive attitudes towards engagement. However, our respondents also reported mostly low levels of engagement at the inform and consult level, highlighting needs and opportunities to advance current practice. These results are critical to inform decisions surrounding the availability of support and resources for health researchers in Manitoba.

## Supplementary information

**Additional file 1.** Survey Questionnaire.

## Data Availability

All reported data are included in the manuscript. Further data are available from the corresponding author on reasonable request.

## Abbreviations

CHI: The George & Fay Yee Centre for Healthcare Innovation
CIHR: Canadian Institutes of Health Research
IAP2: International Association for Public Participation
