# Supplementary material for: Engaging patients and the public in Health Research: experiences, perceptions and training needs among Manitoba health researchers
Source: Res Involv Engagem. 2019 Oct 8;5:28. doi: 10.1186/s40900-019-0162-2 (PMC6781300; doi:10.1186/s40900-019-0162-2)
Supplement: Supplementary file 1 — Additional file 1. Survey Questionnaire. [file 40900_2019_162_MOESM1_ESM.docx]

Additional file 1 **– Survey Questionnaire**

**Eligibility**

- Are you a health researcher who both: (a) spends at least 10% of your working time conducting health research; and (b) holds a faculty appointment at a Manitoba University or who is eligible to apply for competitive health research funding?
- Yes
- No

If YES, you are eligible to complete this survey.

- Which health research pillar do you most identify with?
- Biomedical
- Clinical
- Health systems and services
- Population and public health

**This section asks about your knowledge and experience with engaging patients and/or the public in health research.**

- Have you ever engaged patients and/or the public in your own research?
- Yes
- No

If NO:

- A) What has prevented you for engaging patients and/or the public in your research? Check all that apply.
- Time
- Funding
- Inexperience
- Lack of training
- Lack or organizational directive/mandate to include patients and/or the public
- Not relevant to my research
- Unsure of how to recruit patients and/or the public
- Unsure whether I should be involving patients and/or the public in my research
- Other, please specify….

B) Do you feel you have a good understanding of the field of patient and/or public engagement in health research?

- Yes
- No

If YES:

- Please describe your overall level of *experience* in engaging patients and the public in health research: *(E)*
- A little experience
- Some experience
- Extensive experience
- A) At what level have you engaged patients and/or the public in your research?
- **Inform:** Letting patients/public know about your research findings.
- **Consult:** Obtaining patient/public feedback or input in any component of research.
- **Involve:** Working directly with patients/public throughout the research process to ensure that concerns and aspirations are consistently understood and considered to the maximum extent possible.
- **Collaborate:** To partner with patients/public (i.e. shared decision-making) in each aspect of the research process.
- **Patient-Directed:** Patient/public initiated research where final decision-making of entire research process is in the hands of patients/public.

B) Within the research process, there are numerous opportunities where patients and the public can be engaged. Please indicate those research phases where you have experience engaging patients and the public. (Check all that apply) (E)

- Research priority-setting
- Grant proposal/protocol writing
- Input into methodology/study design
- Development of research questions
- Data collection
- Data analysis
- Interpretation of results
- Input into the selection of knowledge translation products
- Evaluation of research processes
- Determining future research priorities stemming from the results
- Other, please specify….
- Who have you engaged in the research process? Check all that apply (E)
- Patients
- Family/caregivers
- General public
- A geographic community
- Patient/health issue organization
- Community organizations
- Other, please specify…
- How have you recruited patients and the public to engage in research? (E)
- Clinician referrals
- Patient lists (e.g., provided by a clinician)
- Patient organizations
- Advertising through websites
- Advertising through social media and print
- Advertising in mainstream print media
- Advertising in broadcast media
- Local newsletters/papers
- Information kiosks
- Advertisements at medical clinics, health organizations
- Community organizations
- Community leaders
- Cultural/social organizations
- Connected directly (with someone whom I’ve interacted in the past
- I was approached by a patient / member of the public who had an interest in research
- Other: _____________________
- *On a scale of 1 (strongly disagree) to 10 (strongly agree), please rate your agreement with the following statements:*
- I know *how* to engage patients and/or the public in health research. (K)
- With regard to engaging patients and the public in health research, what is expected of me. (K)
- I have the *skills* to engage patients and/or the public in health research. (E)
- I know when it’s appropriate to engage patients and the public in my research. (E)
- Have you participated in training in patient and/or public engagement in health research?
- *Yes, formal certification*
- *Yes, through workshops, seminars, etc.*
- *Yes, informal training (readings, through collaboration on previous projects, etc.)*
- *No*
- *If yes, through what organization?*
- From your perspective, to what extent do current health research activities in your field…

|  | Not at all | A little | Some | A lot | Don’t know |
| --- | --- | --- | --- | --- | --- |
| Include a wide range of patient and/or public perspectives? |  |  |  |  |  |
| Acknowledge patients’ and/or public contributions to research? |  |  |  |  |  |
| Allow patients and/or the public to contribute fully to discussions and decisions? |  |  |  |  |  |
| Acknowledge and value patients’ expertise and experience? |  |  |  |  |  |
| Include patients, researchers, and practitioners working together from the beginning to identify problems and gaps and set priorities for research |  |  |  |  |  |
| Include patients, researchers, and practitioners working together to conduct research? |  |  |  |  |  |

**This section asks about how you perceive patient and public engagement in health research.**

- *Please rate your agreement with the following statements.*

|  | Strongly Disagree | Disagree | Neutral | Agree | Strongly Agree |
| --- | --- | --- | --- | --- | --- |
| In general, I feel that engaging patients and/or the public in health research is important. (A) |  |  |  |  |  |
| I feel that engaging patients and the public in my own research is useful. (A) |  |  |  |  |  |
| I feel that patients and/or the public are actively and meaningfully engaged in health research. (A) |  |  |  |  |  |
| I feel that engaging patients and/or the public in health research can improve the value of research. (A) |  |  |  |  |  |
| I feel that engaging patients and/or the public in health research can improve the healthcare system. (A) |  |  |  |  |  |
| I believe that patients and/or the public have a right to be engaged in health research. (A) |  |  |  |  |  |
| I believe that engaging patients and the public in health research is an integral part of patient oriented research. (A) |  |  |  |  |  |
| I believe that engaging patients and/or the public in my research will interject bias in my research. (A) |  |  |  |  |  |
| I feel pressured to engage patients and the public in my research. (A) |  |  |  |  |  |
| I feel that patients and/or public engagement is compatible with my program of research |  |  |  |  |  |
| I feel that my institution values engaging patients and/or the public in health research |  |  |  |  |  |
| I feel that funding agencies provide sufficient financial reimbursement to researchers for engaging patients and/or the public in health research |  |  |  |  |  |
| I am confident in my ability to involve patients and/or the public in my research |  |  |  |  |  |
| I am comfortable and prepared to explore sensitive topic areas with patients and/or the public |  |  |  |  |  |

- What are some of the barriers you have faced when trying to engage patients and/or the public in your health research (BF). ___________________________________________________________________
- Are there areas of health research where you feel it is inappropriate to engage patients and/or the public? If so, please describe: _____________________________________
- Do you feel that ‘patient engagement’ is an appropriate term?
- Yes
- No
- If no, what term(s) do you use….

**The purpose of this section is to help identify needs and strategies for supporting researchers in involving patients and/or the public in health research (all N).**

- Do you feel that you need support to more effectively engage patients and/or the public in your research?
- Yes
- No

If YES:

- What type/level of support would be most beneficial to you? *Select all that apply.*
- Workshop
- Online module
- Advice/guidance to plan the process
- Support to help find people to engage with in the research process
- Support to facilitate the engagement process over time
- Access to a resource (ie. Website) to let the public know you are seeking public input/partnership
- Manual/guidebook
- Other, please specify…
- Would you participate in patient/public engagement training if it were available?
- Yes
- No

*If YES:*

- What has limited your ability to participate in the past? *Select all that apply*
- Lack of time
- Low priority
- Training not available
- Unsure whether it applies to my research
- Other, please specify: ______________________________
- In which areas listed below would you be interested in receiving additional supports? *Select all that apply*
- What is patient and/or public engagement?
- The rationale for patient and/or public engagement in health research
- Planning for engagement in health research
- Approaches to engagement – surveys, focus groups, advisory groups, social media, etc.
- Recruiting for engagement
- Supporting on-going engagement and addressing challenges to engagement in research
- Engaging specific populations – youth, diverse communities, hard to reach populations, etc.
- Reflexive practice
- Conflict resolution
- Other, please specify: ________________________________

For both YES and No to Question 15:

- If applicable, what are the reasons that you would not be interested in training?
  Select all that apply
- I have already participated in training
- Lack of time
- Low priority
- Lack of organizational support
- Lack of personal interest
- Not relevant to my research
- I feel capable of engaging patients and/or the public in research
- I hire someone to engage patients and the public in my research
- Unsure whether I should be involving patients and/or the public in my research
- N/A
- Other, please specify: _____________________________

**This section asks about you (all D).** *Please answer the following questions.*

- Which of the following best describes your current career stage (D):
- Early-career researcher (5 years or less as an independent researcher)
- Mid-career researcher (6-15 years as an independent researcher)
- Established researcher (16+ years as an independent researcher)
- Which of the following best describes the setting in which you work (D)*:*
- University
- Research Institute
- Hospital
- Other, please specify: ______________________
- What’s your primary research methodology (D)
- Quantitative
- Qualitative
- Mixed methods
- Other, please specify:__________________
- Do you have any additional feedback or comments regarding patient and/or public engagement in Manitoba?

*Legend: K - Knowledge; E - Experience; A - Attitudes; B – Barriers; N – Training Needs; D – Demographics*
